# Glucose-Raising Genetic Variants in *MADD* and *ADCY5* Impair Conversion of Proinsulin to Insulin

**DOI:** 10.1371/journal.pone.0023639

**Published:** 2011-08-22

**Authors:** Robert Wagner, Katarzyna Dudziak, Silke A. Herzberg-Schäfer, Fausto Machicao, Norbert Stefan, Harald Staiger, Hans-Ulrich Häring, Andreas Fritsche

**Affiliations:** 1 Department of Internal Medicine, Division of Endocrinology, Diabetology, Angiology, Nephrology and Clinical Chemistry, Eberhard Karls University Tübingen, Member of the German Center for Diabetes Research (DZD), Tübingen, Germany; 2 Department of Internal Medicine, Division of Nutritional and Preventive Medicine, Tübingen, Germany; Louisiana State University, Pennington Biomedical Research Center, United States of America

## Abstract

**Introduction:**

Recent meta-analyses of genome-wide association studies revealed new genetic loci associated with fasting glycemia. For several of these loci, the mechanism of action in glucose homeostasis is unclear. The objective of the study was to establish metabolic phenotypes for these genetic variants to deliver clues to their pathomechanism.

**Methods:**

In this cross-sectional study 1782 non-diabetic volunteers at increased risk for type 2 diabetes underwent an oral glucose tolerance test. Insulin, C-peptide and proinsulin were measured and genotyping was performed for 12 single nucleotide polymorphisms (SNP) in or near the genes *GCK* (rs4607517), *DGKB* (rs2191349), *GCKR* (rs780094), *ADCY5* (rs11708067), *MADD* (rs7944584), *ADRA2A* (rs10885122), *FADS1* (rs174550), *CRY2* (rs11605924), *SLC2A2* (rs11920090), *PROX1* (rs340874), *GLIS3* (rs7034200) and *C2CD4B* (rs11071657). Parameters of insulin secretion (AUC Insulin_0–30_/AUC Glucose_0–30_, AUC C-peptide_0–120_/AUC Glucose_0–120_), proinsulin-to-insulin conversion (fasting proinsulin, fasting proinsulin/insulin, AUC Proinsulin_0–120_/AUCInsulin_0–120_) and insulin resistance (HOMA-IR, Matsuda-Index) were assessed.

**Results:**

After adjustment for confounding variables, the effect alleles of the *ADCY5* and *MADD* SNPs were associated with an impaired proinsulin-to-insulin conversion (p = 0.002 and p = 0.0001, respectively). *GLIS3* was nominally associated with impaired proinsulin-to-insulin conversion and insulin secretion. The diabetogenic alleles of *DGKB* and *PROX1* were nominally associated with reduced insulin secretion. Nominally significant effects on insulin sensitivity could be found for *MADD* and *PROX1*.

**Discussion:**

By examining parameters of glucose-stimulated proinsulin-to-insulin conversion during an OGTT, we show that the SNP in *ADCY5* is implicated in defective proinsulin-to-insulin conversion. In addition, we confirmed previous findings on the role of a genetic variant in *MADD* on proinsulin-to-insulin conversion. These effects may also be related to neighboring regions of the genome.

## Introduction

Type 2 diabetes is a multifactorial disease that arises from a complex interaction between environmental factors and genetic susceptibility. The major environmental causes are sedentary lifestyle and high energy intake [1]. A multitude of genes contributes to the overall predisposition of type 2 diabetes, but the genes have a rather small individual effect [2]. Most of the approximately 40 known genetic variants conferring increased risk for type 2 diabetes have been discovered by genome-wide association studies (GWAS) [3]. With this method, associations between genomic variants and diabetes prevalence or quantitative glycemic traits like increased fasting glucose (FG) can be established. The variants are single nucleotide polymorphisms (SNPs) harboured in different loci of the genome. Most of the recently discovered SNPs are located in non-coding DNA regions. They may regulate the expression of nearby or far away genes [4], [5], however, the exact target genes and their function in glucose homeostasis are in most cases still elusive. Detailed metabolic characterization of these variants can provide further data to elucidate their mechanism of action.

Hyperglycemia, the sine qua non of diabetes, results from a combination of impaired insulin secretion and impaired insulin action. Although their exact pathophysiologic relevance is unclear, measuring proinsulin, C-peptide and insulin levels can illuminate different aspects of beta-cell dysfunction. In the case of insulin, its level in venous plasma is influenced by extensive hepatic first-pass clearance and a short half-life [6] in combination with pulsatile secretion [7], while C-peptide levels seem to be somewhat more robust [8]. A decreased proinsulin-to-insulin ratio resulting from enhanced proinsulin-to-insulin conversion is supposed to indicate the adaptation of the healthy beta-cell to increased secretory demand after a glucose stimulus [9]. On the other hand, proinsulin-to-insulin ratio increases with age [10] and hyperproinsulinemia has been shown to be an early manifestation of the failing beta cell in prediabetic individuals by prospective investigations [11], [12].

Dynamic glucose metabolism and glucose-stimulated insulin secretion can be further evaluated by observing the above parameters along the course of an oral glucose tolerance test (OGTT). Insulin action can be characterized by insulin sensitivity which can be approximated in several ways [13]–[16].

Nine variants in or near *ADCY5*, *MADD*, *ADRA2A*, *CRY2*, *FADS1*, *PROX1*, *SLC2A2*, *GLIS3*, and *C2CD4B* have been discovered and 7 already known diabetes-related SNPs in *G6PC2*, *MTNR1B*, *GCK*, *DGKB*, *GCKR*, *SLC30A8* and *TCF7L2*, have been confirmed to be associated with FG in a recent meta-analysis [17]. Metabolic phenotypes of the SNPs in *G6PC2* [18], *SLC30A8* [19] and *MTNR1B* [20]–[22] have already been extensively examined by our group and others. We therefore investigated the 12 remaining SNPs in *DGKB*, *GCKR*, *ADCY5*, *MADD*, *FADS1*, *GCK*, *ADRA2A*, *CRY2*, *SLC2A2*, *GLIS3*, *PROX1* and *C2CD4B*. Our objective was to determine novel genotype-phenotype associations.

## Methods

### Subjects

We studied 1782 non-diabetic subjects of European descent with increased risk for type 2 diabetes (positive family history, prior gestational diabetes, glucose intolerance or overweight) who participated in the ongoing Tübingen family study for type 2 diabetes.

All participants underwent a physical examination. Medical history, smoking status and alcohol consumption habits were also obtained. Participants were not taking any medication known to affect glucose tolerance or insulin sensitivity. Metabolic traits were measured during an oral glucose tolerance test. A subgroup of 312 subjects was further characterized by an IVGTT, in part performed within the EUGENE2 network [23]. In addition, hyperinsulinemic-euglycemic clamps were performed in 480 subjects.

Participant characteristics are supplied in Table 1. The ethics committee of the medical faculty of the University of Tübingen approved the study protocol. A written consent was obtained from all participating individuals.

**Table 1:** Clinical characteristics of the study population.

n (NGT^a^/IFG^b^/IGT^c^/IFG+IGT)	1782 (1256/200/172/154)
sex (female/male)	1175/607
age (years)	40±13
BMI (kg/m^2^)	30.0±8.9
Fasting glucose (mmol/l)	5.14±0.55
2-hour-glucose (mmol/l)	6.37±1.64
Fasting insulin (pmol/l)	69.3±58.0
2-hour insulin (pmol/l)	467±487

Data are presented as means±SD.

a normal glucose tolerance.

b impaired fasting glycemia.

c impaired glucose tolerance.

### OGTT, IVGTT and hyperinsulinemic-euglycemic clamp

Following an overnight fast, all participants ingested a 75 g glucose dose at 8 am. Plasma glucose, insulin, proinsulin and C-peptide concentrations were determined after 0, 30, 60, 90 and 120 min. A bedside glucose analyzer (glucose-oxidase method, Yellow Springs Instruments, Yellow Springs, OH, USA) was used to determine plasma glucose.

On a separate occasion, an IVGTT was performed in overnight fasted subjects, as previously described [23]. After baseline samples had been collected, a 0.3 g/kg body weight glucose dose of a 20% glucose solution was given at time 0. Blood samples for the measurement of plasma glucose and insulin were obtained at 2, 4, 6, 8, 10, 20, 30, 40, 50, and 60 min. Hyperinsulinemic-euglycemic clamp was performed, starting at 60 min after the IVGTT glucose bolus or without prior IVGTT. Subjects received a primed infusion of short-acting human insulin (40 mU/m^2^/min) for 120 min. Variable infusion of 20% glucose was started to clamp the plasma glucose concentration at 5 mM. Blood samples for the measurement of plasma glucose were obtained at 5-min intervals. Plasma insulin levels were measured at baseline and at the steady state of the clamp. Plasma glucose and insulin were determined by standard laboratory methods.

Plasma insulin, C-peptide and proinsulin were measured with commercial chemiluminescence assays for ADVIA Centaur (Siemens Medical Solutions, Fernwald, Germany) in accordance with the manufacturer's instructions.

### Genotyping

DNA was extracted from peripheral blood by blood cell lysis, protein precipitation and a washing protocol. All SNPs were genotyped using the MassARRAY platform from Sequenom (Sequenom, San Diego, CA, USA). To verify results, samples of 50 individuals were bidirectionally sequenced for all SNPs using an ABI Prism 310 genetic analyzer (Applied Biosystems). Allele frequencies of all SNPs obeyed Hardy-Weinberg equilibrium and allele frequencies were in accordance with the HapMap CEU Population (Centre d'Etude du Polymorphisme [Utah residents with northern and western European ancestry] Hapmap Data, Phase 1,2 and 3, Release #27).

### Calculations

The AUC for insulin, glucose, C-peptide and proinsulin were calculated with the trapezoid method. AUC Insulin_0–30_/AUC Glucose_0–30_, AUC C-peptide_0–120_/AUC Glucose_0–120_ were calculated to detect effects on insulin secretion as proposed earlier [24]. In this publication we compared 12 indices of insulin release to answer the question which index is best suited to detect genotype effects on insulin secretion. AUC Insulin_0–30_/AUC Glucose_0–30_ and AUC C-peptide_0–120_/AUC Glucose_0–120_ ranked highest. Insulin sensitivity during OGTT was estimated as proposed by Matsuda and DeFronzo [13] [Insulin sensitivity index (ISI-OGTT) = 10,000√(Glu_0_·Ins_0_·Glu_mean_·Ins_mean_)] and by means of the homeostasis model assessment index for insulin resistance (HOMA-IR) [25]. Insulin clearance was calculated as AUC C-peptide_0–120_/AUC Insulin_0–120_. Clamp-derived insulin sensitivity index (ISI-clamp) was calculated as the ratio of the glucose infusion rate necessary to maintain euglycemia during the last 60 min (steady state) of the clamp (in µmol/kg/min) and steady-state insulin concentration (pmol/l).

### Statistical analyses

Variables with non-normal distribution were transformed to their natural logarithms prior to statistical analysis.

Hardy-Weinberg equilibrium was tested using the χ^2^ test. Differences between genotypes were tested with multiple linear regression analysis assuming an additive effect model. All data were adjusted for body mass index (BMI), sex and age. Insulin secretion parameters, proinsulin-to-insulin conversion parameters and proinsulin levels were additionally adjusted for the ISI.

Results with a p<0.05 were considered nominally significant. After correction for multiple testing, i.e. for the investigated SNPs, results with p<0.0042 were considered statistically significant. For the SNP with the highest and lowest minor allele frequencies effect sizes of 0.086 and 0.322, respectively, were detectable with sufficient power (1−ß = 0.8).

Statistical analyses were conducted with JMP 8.0 (SAS Institute, Cary, NC), the power analysis was done with G*Power Ver 3.1.2 [26].

## Results

Key parameters of insulin secretion, proinsulin-to-insulin conversion and insulin sensitivity were investigated after genotyping the cohort of 1782 subjects for 12 SNPs using an additive model. For the SNPs that exhibited effects on proinsulin-to-insulin conversion, we plotted the levels of the investigated blood parameters along the time axis of the OGTT to estimate the dynamic changes after glucose stimulation. For comprehensive data on relevant findings see Table 2, information on all examined SNPs is supplied as supplemental data, see Table S1. In order to attempt a replication of significant findings related to insulin secretion and insulin sensitivity, IVGTT and hyperinsulinemic-euglycemic clamp data were analyzed for the respective SNPs.

**Table 2:** Associations of five SNPs with parameters of glycaemia, insulin sensitivity, insulin secretion and proinsulin-to-insulin conversion (N = 1782).

	ADCY5 rs11708067					MADD rs7944584					DGKB rs2191349					GLIS3 rs7034200					PROX1 rs340874				
	GG^a^	AG^a^	AA^a^	beta (SEM)	*P* d	TT^a^	TA^a^	AA^a^	beta (SEM)	*P* d	GG^a^	GT^a^	TT^a^	beta (SEM)	*P* d	CC^a^	CA^a^	AA^a^	beta (SEM)	*P* d	TT^a^	TC^a^	CC^a^	beta (SEM)	*P* d
N	82	579	1117		.	149	707	893		.	353	877	549		.	464	885	432		.	400	844	530		
Fasting glucose^b^ [mmol/l]	5.01±0.06	5.15±0.02	5.15±0.02	0.0068 (0.0039)	0.0785	5.08±0.05	5.16±0.02	5.13±0.02	0.0019 (0.0035)	0.5951	5.08±0.05	5.16±0.02	5.13±0.02	0.0095 (0.0032)	***0.0027***	5.09±0.02	5.16±0.02	5.16±0.03	0.0077 (0.0032)	0.0147^a^	5.08±0.03	5.17±0.02	5.15±0.03	0.0071 (0.0031)	**0.0228**
2-hour glucose^b^ [mmol/l]	6.09±0.16	6.32±0.07	6.42±0.05	0.0221 (0.0097)	**0.022**	6.33±0.13	6.45±0.06	6.33±0.05	−0.0065 (0.0088)	0.4599	6.33±0.13	6.45±0.06	6.33±0.05	0.0140 (0.0080)	0.0781	6.34±0.08	6.40±0.05	6.35±0.08	0.0009 (0.0079)	0.9067	6.30±0.08	6.37±0.06	6.43±0.07	0.0134 (0.0078)	0.0862
ISI (Matsuda-index)^b^ ^e^ [U]	16.40±1.15	15.29±0.46	15.24±0.31	−0.0352 (0.0212)	0.0967	14.88±0.84	14.88±0.40	15.71±0.35	0.0418 (0.0191)	**0.029**	14.88±0.84	14.88±0.40	15.71±0.35	−0.0244 (0.0174)	0.1625	15.42±0.46	15.11±0.36	15.49±0.51	−0.0132 (0.0173)	0.4459	15.81±0.53	15.10±0.36	15.17±0.45	−0.0359 (0.0170)	**0.0351**
HOMA-IR^b^ ^f^ [U]	2.13±0.21	2.42±0.09	2.33±0.06	0.0361 (0.0207)	0.0821	2.37±0.15	2.43±0.08	2.28±0.07	−0.0514 (0.0188)	**0.0063**	2.37±0.15	2.43±0.08	2.28±0.07	0.0213 (0.0171)	0.2140	2.24±0.09	2.43±0.07	2.31±0.10	0.0133 (0.0169)	0.4315	2.32±0.10	2.42±0.08	2.26±0.08	0.0275 (0.0167)	0.0991
AUC insulin 0–30/AUC glucose 0–30^c^	47.26±3.58	43.53±1.33	44.35±0.98	−0.0127 (0.0165)	0.4418	46.18±3.03	44.03±1.13	43.86±1.09	0.0119 (0.0150)	0.4293	46.18±3.03	44.03±1.13	43.86±1.09	−0.0331 (0.0136)	**0.0149** a	44.02±1.43	45.17±1.10	42.73±1.61	−0.0359 (0.0134)	**0.0075**	45.59±1.67	44.16±1.14	43.38±1.33	−0.0261 (0.0133)	**0.0495**
AUC C-peptide 0–120/AUC glucose 0–120^c^	332.0±13.0	322.6±4.4	323.1±3.3	−0.0069 (0.0115)	0.5471	318.3±9.2	324.1±4.1	324.4±3.6	0.0186 (0.0104)	0.0730	318.3±9.2	324.1±4.1	324.4±3.6	−0.0097 (0.0094)	0.3022	324.4±4.9	325.6±3.6	319.1±5.3	−0.0140 (0.0093)	0.1307	329.1±5.6	323.0±3.8	319.8±4.5	−0.0158 (0.0092)	0.0855
Fasting proinsulin^c^ [pmol/l]	5.23±0.67	5.57±0.29	5.84±0.19	0.0709 (0.0378)	0.0611	5.14±0.64	5.62±0.24	5.86±0.22	0.1089 (0.0340)	**0.0014**	5.14±0.64	5.62±0.24	5.86±0.22	−0.0005 (0.0310)	0.9861	5.75±0.33	5.74±0.22	5.65±0.30	0.0321 (0.0306)	0.2943	5.88±0.36	5.78±0.22	5.52±0.27	0.0018 (0.0304)	0.9519
Fasting proinsulin/fasting insulin^c^	0.110±0.015	0.116±0.010	0.118±0.005	0.0659 (0.0384)	0.0861	0.096±0.015	0.112±0.008	0.121±0.006	0.1258 (0.0344)	***0.0003***	0.096±0.015	0.112±0.008	0.121±0.006	0.0121 (0.0314)	0.6999	0.125±0.012	0.113±0.005	0.113±0.008	0.0364 (0.0310)	0.2404	0.121±0.012	0.118±0.006	0.112±0.007	0.0126 (0.0308)	0.6829
AUC proinsulin 0–120/AUC insulin 0–120^c^	0.040±0.003	0.045±0.002	0.050±0.002	0.0940 (0.0306)	***0.0021***	0.038±0.003	0.048±0.003	0.049±0.001	0.1069 (0.0278)	***0.0001***	0.038±0.003	0.048±0.003	0.049±0.001	−0.0203 (0.0252)	0.4210	0.046±0.002	0.047±0.002	0.053±0.003	0.0616 (0.0249)	**0.0136**	0.044±0.002	0.050±0.002	0.047±0.002	0.0347 (0.0247)	0.1602
AUC proinsulin 60–120/AUC insulin 60–120^c^	0.050±0.005	0.055±0.002	0.063±0.002	0.1078 (0.0320)	***0.0008***	0.046±0.004	0.058±0.003	0.063±0.002	0.1267 (0.0290)	***<0.0001***	0.046±0.004	0.058±0.003	0.063±0.002	−0.0284 (0.0263)	0.2799	0.057±0.003	0.058±0.002	0.065±0.004	0.0602 (0.0261)	**0.0212**	0.057±0.003	0.062±0.003	0.059±0.002	0.0340 (0.0258)	0.1867

Data represent means ± SEM for each genotype.

a Alleles are listed in order of glycemic effect (known glucose-raising allele on the right) and the additive model was applied for all calculations. For the statistical analyses data were log-transformed.

b Plasma glucose levels, ISI and HOMA-IR were adjusted for age, sex and BMI.

c All other parameters were adjusted for age, sex, BMI and ISI.

d P values with nominal significance (<0.05) are indicated with bold, P values significant after correction for multiple testing (<0.0042) are indicated with bold and italic.

e Insulin sensitivity index as proposed by Matsuda and de Fronzo.

f Homeostasis model assessment index for insulin resistance.

The expected association with fasting glucose could be at least nominally confirmed for all but 5 SNPs in the cohort.

**Figure 1:**
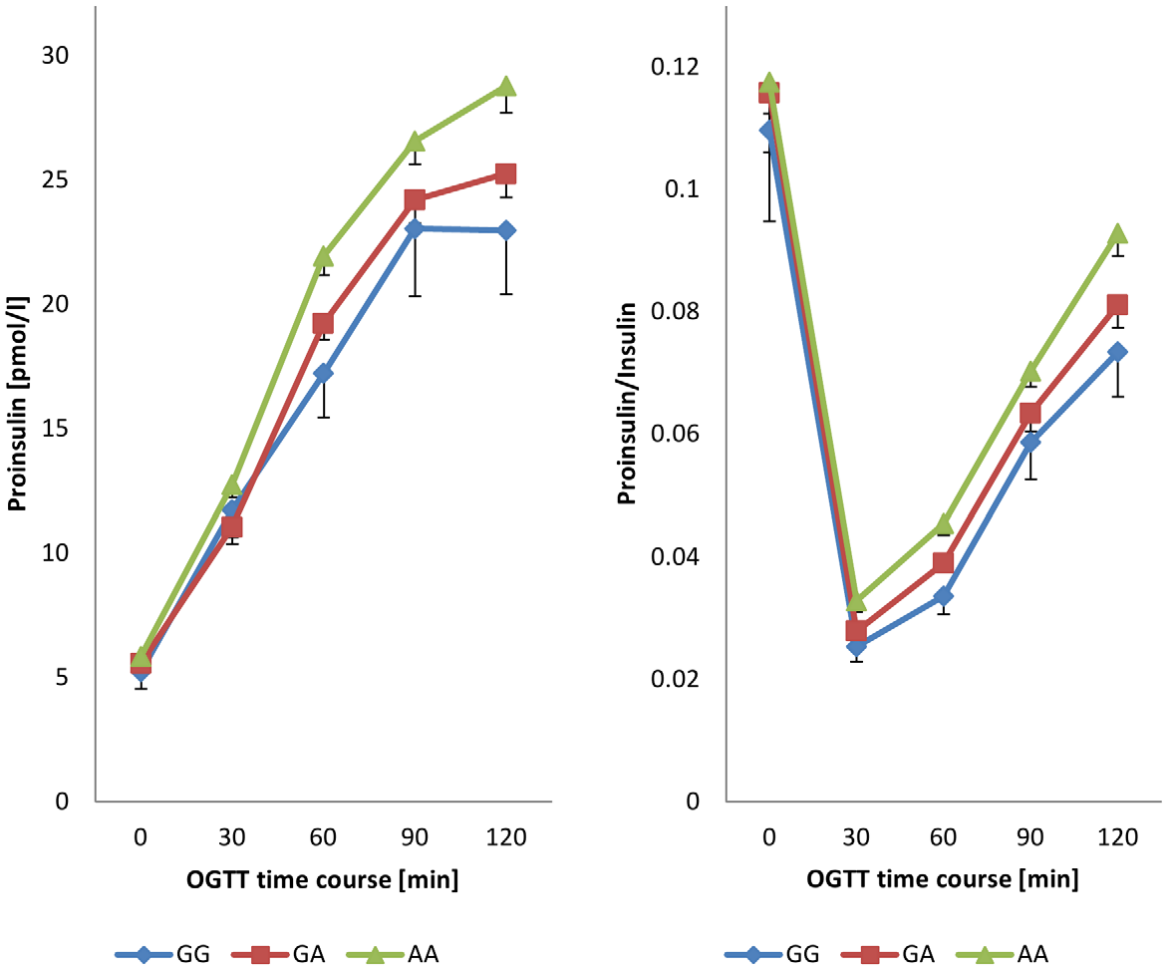
Proinsulin levels and proinsulin-to-insulin ratio during OGTT for genotypes of rs11708067 in *ADCY5*.

We observed effects on **proinsulin-to-insulin conversion** for rs11708067 in *ADCY5* (p = 0.002), rs7944584 in *MADD* (p = 0.0001) and rs7034200 in *GLIS3*, the latter reaching only nominal significance (p = 0.0136). The effect allele in MADD was associated with an elevation of the proinsulin-insulin ratio at all time-points tested, implicating impaired proinsulin-to-insulin conversion. The other two SNPs associated with impaired glucose-stimulated proinsulin-to-insulin conversion only. However, in the case of both *MADD* and *ADCY5* (Figure 1), the effect size tended to be larger for the integrated and late-stage OGTT-derived proinsulin-to-insulin conversion parameters.

No effect on **insulin secretion** could be seen for rs7944584 in *MADD* and rs11708067 in *ADCY5*, but rs7034200 in *GLIS3* had a nominally significant association with insulin secretion (AUC Insulin_0–30_/AUC Glucose_0–30_, p = 0.0075). Furthermore, glucose-stimulated insulin secretion was nominally associated with rs340874 in *PROX1* (p = 0.0495) and rs2191349 in *DGKB* (p = 0.0149). AUC insulin_0–10_ levels during the IVGTT showed no significant associations with these SNPs (p = 0.11 for DGKB, p = 0.45 for PROX1 and p = 0.8 for GLIS3).

As for **insulin sensitivity**, nominally significant associations could be found for rs340874 in *PROX1* (ISI-OGTT, p = 0.0351) and rs7944584 in *MADD* (ISI-OGTT, p = 0.029 and HOMA-IR, p = 0.0063). The clamp-derived insulin sensitivity index did not associate with these SNPs (ISI-clamp, p>0.65).

Further nominally significant associations were observed for rs11920090 in *SLC2A2* and rs10885122 in *ADRA2A* with insulin sensitivity and fasting proinsulin-to-insulin conversion, respectively, but the minor allele frequencies were too low (14% and 12%, respectively) to establish plausible relationships.

Insulin clearance showed a nominally significant association (p = 0.048) with the genotypes in PROX1.

## Discussion

We tested 12 newly discovered glucose-raising genetic variants in an extensively phenotyped cohort of non-diabetic subjects to identify the mechanism of action leading to increased fasting glucose.

The findings of Ingelsson et al [27] on impaired proinsulin-to-insulin conversion could be fully replicated for the SNP in the *MADD* gene. The level of significance was convincingly strong not only for the fasting proinsulin-to-insulin-ratio, but for the proinsulin-to-insulin conversion parameters throughout the OGTT as well. Similarly strong associations were established for glucose-stimulated proinsulin-to-insulin conversion in the case of the *ADCY5* variant. This effect allele did not relate to the fasting proinsulin-to-insulin ratio though; in fact differences between the genotypes became more prominent after the 1^st^ hour of the OGTT. An effect on proinsulin-to-insulin conversion was suggested for the examined SNP in *GLIS3* as well, yet we could not show it at the required level of significance. Since a relevant proportion (326 out of 1782) of the participants turned out to have impaired glucose tolerance (IGT) which could confound proinsulin-to-insulin conversion [11], [28], we also tested the influence of this effect. Further adjustment on IGT did not change the findings (data not shown).

How these variants influence proinsulin-to-insulin conversion is unclear. The examined SNPs are named after the closest gene, but linkage disequilibrium with other, mostly nearby, parts of the genome could easily hide a different SNP as a causative agent for the established associations. For example, *MADD*, an acronym of mitogen-activated protein kinase (MAPK) activating death domain, encodes an adaptor protein that plays a role in MAPK activation. However, no plausible link to proinsulin-to-insulin conversion could be found in the literature for this gene. Several neighboring genes have been discussed as potential candidates instead [27], [29]. Of these, genes that encode proteins involved in vesicle trafficking between the endoplasmic reticulum (ER) and the Golgi-apparatus could provide an enlightening hypothesis on the findings.

The gene product of *ADCY5* is a member of membrane-bound adenylyl-cyclase enzymes, which is most abundantly expressed in the heart and brain, but also present in pancreatic beta cells. It catalyzes the production of cAMP, which can modulate glucose-stimulated insulin secretion by several possible mechanisms [30]. Since knocking out the *ADCY5* gene in mice may protect cardiac muscle from apoptosis [31], a similar effect on beta cell apoptosis and thus beta cell mass has been postulated [32]. Nonetheless, the presently demonstrated solitary association of the *ADCY5* variant with proinsulin-to-insulin conversion does not fit neatly to these hypothetical ways of action. Interestingly, the investigated SNP in *ADCY5* could also serve as a proxy for neighboring genes involved in protein folding and vesicle trafficking. A large linkage block spanning about 260,000 base pairs comprises the investigated rs11708067 in *ADCY5*, the whole *SEC22* gene and a part of the *PDIA5* gene. *SEC22A* encodes a protein belonging to the SEC22 family of vesicle trafficking proteins [33]. Protein disulfide isomerase family A, member 5 (PDIA5) belongs to a family of enzymes that mediate oxidative protein folding in the endoplasmic reticulum [34].

Dysfunctional regulatory pathways in the transport mechanism and protein folding machinery can lead to an increase in misfolded proteins in the ER and Golgi-apparatus [35]. Molecular overcrowding further impairs correct folding of the polypeptide chains resulting in a dramatically increased accumulation of misfolded proinsulin when secretory demand rises [36]. Therefore, more proinsulin is released from the beta cell [35].

The genetic variation in *MADD* and *ADCY5* did not affect insulin secretion neither in our population nor in the study of Ingelsson et al. This leads to the hypothesis that an impaired conversion of proinsulin to insulin mirrors beta cell dysfunction, which is sufficient to increase glucose levels. Minor effects on insulin secretion may have been missed due to the relatively small number of subjects in the present study. Effects on insulin secretion have to be established in future studies. However, we were able to detect possible effects on insulin secretion for SNPs in *DGKB*, *GLIS3* and *PROX1* which could not withstand correction for multiple testing though. These findings failed replication by IVGTT, and this may be due to the lack of sufficient statistical power in the smaller subgroup.

Our findings suggest an association of the effect allele in *MADD* with insulin sensitivity, but it is unexpectedly a positive correlation conferring improved insulin sensitivity for the variant with higher fasting glucose. Due to the marked effect of the risk allele in *MADD* on conversion of proinsulin to insulin, the fasting insulin levels were significantly reduced in carriers of the risk allele (see Table S1). Therefore, the improved insulin sensitivity suggested by the used indices may result only from the calculation at a markedly impaired proinsulin-to-insulin conversion and probably does not reflect a true effect on insulin sensitivity. It is of note that we could not replicate this effect of the *MADD* effect allele on insulin sensitivity using the gold standard method of the euglycemic hyperinsulinemic clamp. This also argues against a true effect observed with the OGTT derived insulin sensitivity data.

The present study has certain limitations that need to be taken into account. The cohort of this study includes only subjects of European descent, and the results therefore cannot be generalized. Moreover, the cross-sectional nature of the study and the lack of replication in a different cohort have to be noted.

In conclusion, we demonstrated an effect of rs11708067 in *ADCY5* on proinsulin-to-insulin conversion. Because there are other genes in the adjacent region of the genome that could pathogenetically more plausibly explain the association with proinsulin-to-insulin conversion, exploring further SNPs in this region may reveal stronger associations with proinsulin-to-insulin conversion in the future.

## Supporting Information

Table S1 **Association of all investigated SNPs (** ***ADCY5*** **rs11708067,** ***MADD*** **rs7944584,** ***GCK*** **rs4607517,** ***DGKB*** **rs2191349,** ***GCKR*** **rs780094,** ***ADRA2A*** **rs10885122,** ***FADS1*** **rs174550,** ***CRY2*** **rs11605924,** ***SLC2A2*** **rs11920090,** ***GLIS3*** **rs7034200,** ***PROX1*** **rs340874,** ***C2CD4B*** **rs11071657) with parameters of glycemia, insulin sensitivity, insulin secretion and proinsulin-to-insulin conversion.** Mean values and their standard errors are listed for each genotype. Effect sizes are provided as beta (± standard error). For the linear regression analysis, data were log-transformed. Plasma glucose levels, ISI and HOMA-IR were adjusted for age, sex and BMI. All other parameters were adjusted for age, sex, BMI and ISI.
(DOC)
