# Supplementary material for: Glucose-Raising Genetic Variants in MADD and ADCY5 Impair Conversion of Proinsulin to Insulin
Source: PLoS One. 2011 Aug 22;6(8):e23639. doi: 10.1371/journal.pone.0023639 (PMC3161735; doi:10.1371/journal.pone.0023639)
Supplement: Table S1 — Association of all investigated SNPs ( ADCY5 rs11708067, MADD rs7944584, GCK rs4607517, DGKB rs2191349, GCKR rs780094, ADRA2A rs10885122, FADS1 rs174550, CRY2 rs11605924, SLC2A2 rs11920090, GLIS3 rs7034200, PROX1 rs340874, C2CD4B rs11071657) with parameters of glycemia, insulin sensitivity, insulin secretion and proinsulin-to-insulin conversion. Mean values and their standard errors are listed for each genotype. Effect sizes are provided as beta (± standard error). For the linear regression analysis, data were log-transformed. Plasma glucose levels, ISI and HOMA-IR were adjusted for age, sex and BMI. All other parameters were adjusted for age, sex, BMI and ISI. (DOC) [file pone.0023639.s001.doc]

|  | GG | GA | AA | GG | GA | AA |  |  |  |
| --- | --- | --- | --- | --- | --- | --- | --- | --- | --- |
| **ADCY5 rs11708067** | 82 | 579 | 1117 |  |  |  |  |  |  |
| Glucose 0 [mmol/l] | 5.005 | 5.151 | 5.146 | 0.057 | 0.023 | 0.017 | 0.007 | 0.004 | 0.079 |
| Glucose 120 [mmol/l] | 6.091 | 6.316 | 6.420 | 0.160 | 0.066 | 0.050 | 0.022 | 0.010 | 0.022 |
| Insulin 0 [pmol/l] | 65.122 | 71.164 | 68.600 | 6.082 | 2.602 | 1.664 | 0.029 | 0.019 | 0.132 |
| ISI-Matsuda [U/l] | 16.397 | 15.294 | 15.243 | 1.147 | 0.460 | 0.307 | -0.035 | 0.021 | 0.097 |
| HOMA-IR [U] | 2.1261 | 2.4161 | 2.3287 | 0.2102 | 0.0941 | 0.0612 | 0.0361 | 0.0207 | 0.0821 |
| AUC insulin 0-30/AUC glucose 0-30 | 47.263 | 43.527 | 44.355 | 3.582 | 1.331 | 0.977 | -0.013 | 0.017 | 0.442 |
| Insulinogenic Index | 159.628 | 136.725 | 154.304 | 11.604 | 17.659 | 6.056 | -0.002 | 0.026 | 0.948 |
| AUC C-peptide 0-30/AUC glucose 0-30 | 209.311 | 202.087 | 205.771 | 8.759 | 3.244 | 2.471 | 0.002 | 0.012 | 0.845 |
| AUC C-peptide 0-120/AUC glucose 0-120 | 331.987 | 322.582 | 323.099 | 13.019 | 4.364 | 3.259 | -0.007 | 0.011 | 0.547 |
| Insulin 30 [pmol/] | 576.852 | 530.572 | 537.605 | 50.538 | 17.798 | 12.504 | -0.013 | 0.017 | 0.460 |
| Proinsulin 0 [pmol/l] | 5.227 | 5.568 | 5.843 | 0.671 | 0.290 | 0.190 | 0.071 | 0.038 | 0.061 |
| Proinsulin 0/Insulin 0 | 0.1097 | 0.1158 | 0.1175 | 0.0148 | 0.0097 | 0.0051 | 0.0659 | 0.0384 | 0.0861 |
| AUC proinsulin 0-120/AUC insulin 0-120 | 0.0401 | 0.0446 | 0.0502 | 0.0034 | 0.0016 | 0.0019 | 0.0940 | 0.0306 | 0.0021 |
| AUC proinsulin 60-120/AUC insulin 60-120 | 0.0502 | 0.0553 | 0.0625 | 0.0045 | 0.0020 | 0.0022 | 0.1078 | 0.0320 | 0.0008 |
|  |  |  |  |  |  |  |  |  |  |
|  | TT | TA | AA | TT | TA | AA |  |  |  |
| **MADD rs7944584** | 149 | 707 | 893 |  |  |  |  |  |  |
| Glucose 0 [mmol/l] | 5.078 | 5.162 | 5.133 | 0.047 | 0.021 | 0.018 | 0.002 | 0.004 | 0.595 |
| Glucose 120 [mmol/l] | 6.331 | 6.446 | 6.327 | 0.131 | 0.064 | 0.054 | -0.006 | 0.009 | 0.460 |
| Insulin 0 [pmol/l] | 71.722 | 71.288 | 67.488 | 4.382 | 2.215 | 1.945 | -0.053 | 0.018 | 0.003 |
| ISI-Matsuda [U/l] | 14.878 | 14.877 | 15.712 | 0.840 | 0.400 | 0.352 | 0.042 | 0.019 | 0.029 |
| HOMA-IR [U] | 2.3668 | 2.4310 | 2.2847 | 0.1467 | 0.0810 | 0.0715 | -0.0514 | 0.0188 | 0.0063 |
| AUC insulin 0-30/AUC glucose 0-30 | 46.182 | 44.032 | 43.861 | 3.027 | 1.132 | 1.090 | 0.012 | 0.015 | 0.429 |
| Insulinogenic Index | 164.123 | 138.888 | 153.674 | 13.198 | 14.313 | 7.440 | 0.026 | 0.023 | 0.261 |
| AUC C-peptide 0-30/AUC glucose 0-30 | 206.117 | 205.620 | 204.271 | 7.237 | 3.006 | 2.695 | 0.009 | 0.011 | 0.420 |
| AUC C-peptide 0-120/AUC glucose 0-120 | 318.298 | 324.112 | 324.400 | 9.238 | 4.051 | 3.610 | 0.019 | 0.010 | 0.073 |
| Insulin 30 [pmol/] | 539.949 | 539.640 | 530.953 | 35.705 | 15.015 | 14.243 | 0.023 | 0.016 | 0.149 |
| Proinsulin 0 [pmol/l] | 5.136 | 5.620 | 5.857 | 0.639 | 0.238 | 0.215 | 0.109 | 0.034 | 0.001 |
| Proinsulin 0/Insulin 0 | 0.0955 | 0.1121 | 0.1209 | 0.0149 | 0.0079 | 0.0056 | 0.1258 | 0.0344 | 0.0003 |
| AUC proinsulin 0-120/AUC insulin 0-120 | 0.0384 | 0.0478 | 0.0494 | 0.0035 | 0.0027 | 0.0014 | 0.1069 | 0.0278 | 0.0001 |
| AUC proinsulin 60-120/AUC insulin 60-120 | 0.0463 | 0.0582 | 0.0629 | 0.0037 | 0.0029 | 0.0019 | 0.1267 | 0.0290 | 0.0000 |
|  |  |  |  |  |  |  |  |  |  |
|  | GG | GA | AA | GG | GA | AA |  |  |  |
| **GCK rs4607517** | 1150 | 500 | 77 |  |  |  |  |  |  |
| Glucose 0 [mmol/l] | 5.124 | 5.166 | 5.226 | 0.016 | 0.025 | 0.071 | 0.012 | 0.004 | 0.003 |
| Glucose 120 [mmol/l] | 6.354 | 6.396 | 6.605 | 0.049 | 0.072 | 0.190 | 0.020 | 0.010 | 0.048 |
| Insulin 0 [pmol/l] | 70.661 | 67.433 | 65.241 | 1.788 | 2.375 | 5.705 | 0.007 | 0.020 | 0.737 |
| ISI-Matsuda [U/l] | 15.379 | 15.462 | 13.940 | 0.319 | 0.469 | 0.928 | -0.026 | 0.022 | 0.233 |
| HOMA-IR [U] | 2.3799 | 2.3087 | 2.2609 | 0.0642 | 0.0887 | 0.2233 | 0.0184 | 0.0214 | 0.3907 |
| AUC insulin 0-30/AUC glucose 0-30 | 45.530 | 41.917 | 42.516 | 1.016 | 1.277 | 3.306 | -0.027 | 0.017 | 0.119 |
| Insulinogenic Index | 150.244 | 152.550 | 114.778 | 10.059 | 7.067 | 27.820 | -0.039 | 0.026 | 0.138 |
| AUC C-peptide 0-30/AUC glucose 0-30 | 207.856 | 200.768 | 198.413 | 2.516 | 3.355 | 7.254 | -0.015 | 0.013 | 0.226 |
| AUC C-peptide 0-120/AUC glucose 0-120 | 326.753 | 319.598 | 312.601 | 3.319 | 4.695 | 8.447 | -0.015 | 0.012 | 0.195 |
| Insulin 30 [pmol/] | 550.416 | 511.038 | 530.996 | 13.098 | 16.822 | 47.734 | -0.018 | 0.018 | 0.312 |
| Proinsulin 0 [pmol/l] | 5.980 | 5.603 | 4.316 | 0.209 | 0.260 | 0.390 | -0.056 | 0.039 | 0.145 |
| Proinsulin 0/Insulin 0 | 0.1239 | 0.1123 | 0.0796 | 0.0065 | 0.0063 | 0.0071 | -0.0463 | 0.0391 | 0.2366 |
| AUC proinsulin 0-120/AUC insulin 0-120 | 0.0494 | 0.0470 | 0.0399 | 0.0018 | 0.0018 | 0.0030 | -0.0086 | 0.0315 | 0.7843 |
| AUC proinsulin 60-120/AUC insulin 60-120 | 0.0609 | 0.0597 | 0.0507 | 0.0021 | 0.0025 | 0.0041 | 0.0038 | 0.0330 | 0.9090 |

|  |  |  |  |  |  |  |  |  |  |
| --- | --- | --- | --- | --- | --- | --- | --- | --- | --- |
|  | GG | GT | TT | GG | GT | TT |  |  |  |
| **DGKB rs2191349** | 353 | 877 | 549 |  |  |  |  |  |  |
| Glucose 0 [mmol/l] | 5.078 | 5.152 | 5.165 | 0.028 | 0.019 | 0.024 | 0.010 | 0.003 | 0.003 |
| Glucose 120 [mmol/l] | 6.258 | 6.402 | 6.396 | 0.084 | 0.056 | 0.070 | 0.014 | 0.008 | 0.078 |
| Insulin 0 [pmol/l] | 68.937 | 71.145 | 66.681 | 3.175 | 2.018 | 2.299 | 0.012 | 0.016 | 0.464 |
| ISI-Matsuda [U/l] | 15.365 | 15.097 | 15.604 | 0.543 | 0.362 | 0.447 | -0.024 | 0.017 | 0.162 |
| HOMA-IR [U] | 2.2958 | 2.4155 | 2.2799 | 0.1140 | 0.0732 | 0.0857 | 0.0213 | 0.0171 | 0.2140 |
| AUC insulin 0-30/AUC glucose 0-30 | 45.320 | 45.043 | 42.230 | 1.698 | 1.138 | 1.306 | -0.033 | 0.014 | 0.015 |
| Insulinogenic Index | 166.134 | 148.827 | 137.739 | 10.467 | 11.822 | 9.976 | -0.043 | 0.021 | 0.042 |
| AUC C-peptide 0-30/AUC glucose 0-30 | 207.565 | 205.060 | 202.428 | 3.980 | 2.801 | 3.479 | -0.015 | 0.010 | 0.155 |
| AUC C-peptide 0-120/AUC glucose 0-120 | 325.260 | 324.379 | 320.490 | 5.463 | 3.741 | 4.564 | -0.010 | 0.009 | 0.302 |
| Insulin 30 [pmol/] | 546.091 | 548.460 | 513.689 | 21.916 | 14.914 | 17.013 | -0.031 | 0.014 | 0.029 |
| Proinsulin 0 [pmol/l] | 5.477 | 5.721 | 5.888 | 0.310 | 0.205 | 0.327 | -0.001 | 0.031 | 0.986 |
| Proinsulin 0/Insulin 0 | 0.1090 | 0.1125 | 0.1279 | 0.0070 | 0.0058 | 0.0106 | 0.0121 | 0.0314 | 0.6999 |
| AUC proinsulin 0-120/AUC insulin 0-120 | 0.0506 | 0.0464 | 0.0482 | 0.0037 | 0.0018 | 0.0019 | -0.0203 | 0.0252 | 0.4210 |
| AUC proinsulin 60-120/AUC insulin 60-120 | 0.0633 | 0.0578 | 0.0600 | 0.0041 | 0.0021 | 0.0025 | -0.0284 | 0.0263 | 0.2799 |
|  |  |  |  |  |  |  |  |  |  |
|  | TT | TC | CC | TT | TC | CC |  |  |  |
| **GCKR rs780094** | 330 | 866 | 563 |  |  |  |  |  |  |
| Glucose 0 [mmol/l] | 5.064 | 5.128 | 5.203 | 0.028 | 0.019 | 0.024 | 0.012 | 0.003 | 0.000 |
| Glucose 120 [mmol/l] | 6.460 | 6.337 | 6.371 | 0.091 | 0.057 | 0.067 | -0.007 | 0.008 | 0.406 |
| Insulin 0 [pmol/l] | 64.242 | 70.004 | 71.070 | 2.683 | 2.043 | 2.472 | 0.016 | 0.016 | 0.329 |
| ISI-Matsuda [U/l] | 15.679 | 15.515 | 14.717 | 0.552 | 0.377 | 0.413 | -0.018 | 0.018 | 0.313 |
| HOMA-IR [U] | 2.1185 | 2.3670 | 2.4475 | 0.0922 | 0.0736 | 0.0932 | 0.0275 | 0.0172 | 0.1107 |
| AUC insulin 0-30/AUC glucose 0-30 | 42.853 | 44.648 | 44.351 | 1.784 | 1.113 | 1.348 | -0.015 | 0.014 | 0.280 |
| Insulinogenic Index | 119.447 | 156.633 | 152.759 | 29.798 | 5.896 | 9.372 | -0.011 | 0.021 | 0.609 |
| AUC C-peptide 0-30/AUC glucose 0-30 | 196.180 | 207.499 | 205.737 | 4.531 | 2.662 | 3.570 | 0.007 | 0.010 | 0.484 |
| AUC C-peptide 0-120/AUC glucose 0-120 | 308.602 | 328.190 | 325.591 | 5.826 | 3.603 | 4.738 | 0.013 | 0.010 | 0.180 |
| Insulin 30 [pmol/] | 511.445 | 543.768 | 540.951 | 22.512 | 14.604 | 17.716 | -0.009 | 0.015 | 0.541 |
| Proinsulin 0 [pmol/l] | 5.215 | 5.716 | 6.009 | 0.327 | 0.220 | 0.293 | 0.040 | 0.031 | 0.203 |
| Proinsulin 0/Insulin 0 | 0.1052 | 0.1182 | 0.1212 | 0.0071 | 0.0061 | 0.0099 | 0.0364 | 0.0318 | 0.2520 |
| AUC proinsulin 0-120/AUC insulin 0-120 | 0.0455 | 0.0511 | 0.0444 | 0.0022 | 0.0024 | 0.0014 | 0.0080 | 0.0256 | 0.7540 |
| AUC proinsulin 60-120/AUC insulin 60-120 | 0.0569 | 0.0638 | 0.0549 | 0.0028 | 0.0027 | 0.0018 | -0.0043 | 0.0267 | 0.8708 |
|  |  |  |  |  |  |  |  |  |  |
|  | TT | TG | GG | TT | TG | GG |  |  |  |
| **ADRA2A rs10885122** | 32 | 354 | 1392 |  |  |  |  |  |  |
| Glucose 0 [mmol/l] | 5.447 | 5.087 | 5.148 | 0.108 | 0.028 | 0.015 | 0.002 | 0.005 | 0.740 |
| Glucose 120 [mmol/l] | 6.753 | 6.222 | 6.404 | 0.263 | 0.088 | 0.044 | 0.013 | 0.012 | 0.269 |
| Insulin 0 [pmol/l] | 88.758 | 67.529 | 69.355 | 14.943 | 2.882 | 1.557 | -0.022 | 0.024 | 0.357 |
| ISI-Matsuda [U/l] | 12.236 | 15.639 | 15.262 | 1.583 | 0.585 | 0.279 | 0.031 | 0.026 | 0.234 |
| HOMA-IR [U] | 3.2042 | 2.2455 | 2.3566 | 0.5776 | 0.1014 | 0.0570 | -0.0208 | 0.0259 | 0.4223 |
| AUC insulin 0-30/AUC glucose 0-30 | 52.307 | 44.910 | 43.930 | 6.683 | 1.814 | 0.854 | -0.024 | 0.021 | 0.249 |
| Insulinogenic Index | 168.036 | 166.472 | 144.000 | 23.536 | 8.914 | 8.517 | -0.032 | 0.032 | 0.316 |
| AUC C-peptide 0-30/AUC glucose 0-30 | 222.946 | 205.694 | 204.291 | 16.543 | 4.377 | 2.163 | -0.014 | 0.015 | 0.376 |
| AUC C-peptide 0-120/AUC glucose 0-120 | 337.251 | 328.153 | 322.186 | 18.687 | 5.935 | 2.887 | -0.014 | 0.014 | 0.337 |
| Insulin 30 [pmol/] | 678.374 | 546.784 | 532.331 | 87.418 | 23.818 | 11.108 | -0.031 | 0.022 | 0.156 |
| Proinsulin 0 [pmol/l] | 7.226 | 5.798 | 5.674 | 1.060 | 0.344 | 0.177 | -0.098 | 0.047 | 0.037 |
| Proinsulin 0/Insulin 0 | 0.1368 | 0.1203 | 0.1151 | 0.0312 | 0.0092 | 0.0053 | -0.0971 | 0.0475 | 0.0409 |
| AUC proinsulin 0-120/AUC insulin 0-120 | 0.0802 | 0.0479 | 0.0471 | 0.0338 | 0.0022 | 0.0013 | -0.0377 | 0.0380 | 0.3219 |
| AUC proinsulin 60-120/AUC insulin 60-120 | 0.0921 | 0.0608 | 0.0585 | 0.0336 | 0.0031 | 0.0016 | -0.0441 | 0.0397 | 0.2671 |

|  |  |  |  |  |  |  |  |  |  |
| --- | --- | --- | --- | --- | --- | --- | --- | --- | --- |
|  | CC | CT | TT | CC | CT | TT |  |  |  |
| **FADS1 rs174550** | 151 | 784 | 823 |  |  |  |  |  |  |
| Glucose 0 [mmol/l] | 5.189 | 5.150 | 5.121 | 0.048 | 0.020 | 0.019 | -0.003 | 0.004 | 0.352 |
| Glucose 120 [mmol/l] | 6.145 | 6.383 | 6.413 | 0.142 | 0.057 | 0.058 | 0.017 | 0.009 | 0.053 |
| Insulin 0 [pmol/l] | 68.058 | 70.629 | 67.379 | 4.133 | 2.134 | 1.894 | -0.025 | 0.018 | 0.165 |
| ISI-Matsuda [U/l] | 15.841 | 14.865 | 15.632 | 0.862 | 0.364 | 0.378 | 0.014 | 0.019 | 0.473 |
| HOMA-IR [U] | 2.3330 | 2.4094 | 2.2600 | 0.1523 | 0.0796 | 0.0672 | -0.0278 | 0.0189 | 0.1400 |
| AUC insulin 0-30/AUC glucose 0-30 | 43.887 | 44.885 | 43.275 | 2.610 | 1.154 | 1.113 | -0.019 | 0.015 | 0.216 |
| Insulinogenic Index | 156.990 | 145.483 | 148.629 | 10.094 | 14.315 | 5.741 | -0.028 | 0.023 | 0.230 |
| AUC C-peptide 0-30/AUC glucose 0-30 | 201.089 | 205.616 | 204.223 | 6.659 | 2.876 | 2.836 | 0.003 | 0.011 | 0.813 |
| AUC C-peptide 0-120/AUC glucose 0-120 | 316.373 | 326.452 | 321.736 | 9.015 | 3.938 | 3.703 | 0.003 | 0.010 | 0.768 |
| Insulin 30 [pmol/] | 539.335 | 548.475 | 521.373 | 34.175 | 15.354 | 14.288 | -0.024 | 0.016 | 0.123 |
| Proinsulin 0 [pmol/l] | 5.862 | 5.784 | 5.678 | 0.595 | 0.217 | 0.239 | -0.028 | 0.034 | 0.411 |
| Proinsulin 0/Insulin 0 | 0.1330 | 0.1140 | 0.1173 | 0.0288 | 0.0059 | 0.0061 | -0.0146 | 0.0348 | 0.6744 |
| AUC proinsulin 0-120/AUC insulin 0-120 | 0.0545 | 0.0483 | 0.0466 | 0.0080 | 0.0020 | 0.0014 | -0.0399 | 0.0281 | 0.1557 |
| AUC proinsulin 60-120/AUC insulin 60-120 | 0.0677 | 0.0603 | 0.0579 | 0.0082 | 0.0024 | 0.0018 | -0.0468 | 0.0293 | 0.1105 |
|  |  |  |  |  |  |  |  |  |  |
|  | CC | CA | AA | CC | CA | AA |  |  |  |
| **CRY2 rs11605924** | 478 | 883 | 411 |  |  |  |  |  |  |
| Glucose 0 [mmol/l] | 5.098 | 5.133 | 5.208 | 0.026 | 0.019 | 0.025 | 0.010 | 0.003 | 0.001 |
| Glucose 120 [mmol/l] | 6.396 | 6.329 | 6.454 | 0.071 | 0.055 | 0.085 | -0.002 | 0.008 | 0.779 |
| Insulin 0 [pmol/l] | 69.214 | 69.795 | 68.907 | 2.715 | 1.922 | 2.888 | -0.011 | 0.016 | 0.482 |
| ISI-Matsuda [U/l] | 15.177 | 15.594 | 14.645 | 0.466 | 0.374 | 0.467 | 0.004 | 0.017 | 0.811 |
| HOMA-IR [U] | 2.3251 | 2.3713 | 2.3454 | 0.0980 | 0.0713 | 0.1028 | -0.0009 | 0.0170 | 0.9600 |
| AUC insulin 0-30/AUC glucose 0-30 | 45.521 | 44.013 | 43.481 | 1.569 | 1.082 | 1.523 | -0.015 | 0.013 | 0.254 |
| Insulinogenic Index | 143.823 | 150.900 | 150.086 | 21.608 | 7.057 | 5.793 | 0.001 | 0.021 | 0.969 |
| AUC C-peptide 0-30/AUC glucose 0-30 | 210.281 | 203.798 | 202.046 | 3.939 | 2.686 | 3.871 | -0.017 | 0.010 | 0.091 |
| AUC C-peptide 0-120/AUC glucose 0-120 | 329.326 | 322.949 | 319.354 | 5.095 | 3.612 | 5.246 | -0.015 | 0.009 | 0.114 |
| Insulin 30 [pmol/] | 553.688 | 533.489 | 530.108 | 20.526 | 14.341 | 19.006 | -0.010 | 0.014 | 0.477 |
| Proinsulin 0 [pmol/l] | 5.471 | 6.100 | 5.201 | 0.294 | 0.238 | 0.266 | 0.006 | 0.031 | 0.844 |
| Proinsulin 0/Insulin 0 | 0.1083 | 0.1253 | 0.1067 | 0.0071 | 0.0076 | 0.0073 | 0.0132 | 0.0312 | 0.6736 |
| AUC proinsulin 0-120/AUC insulin 0-120 | 0.0482 | 0.0483 | 0.0456 | 0.0030 | 0.0019 | 0.0019 | 0.0106 | 0.0251 | 0.6728 |
| AUC proinsulin 60-120/AUC insulin 60-120 | 0.0594 | 0.0601 | 0.0576 | 0.0033 | 0.0022 | 0.0026 | 0.0055 | 0.0263 | 0.8350 |
|  |  |  |  |  |  |  |  |  |  |
|  | AA | AT | TT | AA | AT | TT |  |  |  |
| **SLC2A2 rs11920090** | 36 | 433 | 1306 |  |  |  |  |  |  |
| Glucose 0 [mmol/l] | 5.121 | 5.093 | 5.158 | 0.097 | 0.026 | 0.015 | 0.011 | 0.005 | 0.018 |
| Glucose 120 [mmol/l] | 6.500 | 6.275 | 6.405 | 0.331 | 0.076 | 0.046 | 0.013 | 0.011 | 0.259 |
| Insulin 0 [pmol/l] | 64.392 | 69.338 | 69.523 | 12.490 | 2.863 | 1.577 | 0.022 | 0.023 | 0.346 |
| ISI-Matsuda [U/l] | 18.534 | 15.713 | 15.040 | 2.061 | 0.504 | 0.289 | -0.049 | 0.025 | 0.047 |
| HOMA-IR [U] | 2.2011 | 2.3230 | 2.3644 | 0.4740 | 0.1008 | 0.0581 | 0.0322 | 0.0243 | 0.1856 |
| AUC insulin 0-30/AUC glucose 0-30 | 36.993 | 43.485 | 44.728 | 4.430 | 1.720 | 0.870 | 0.024 | 0.019 | 0.219 |
| Insulinogenic Index | 134.360 | 131.163 | 154.737 | 17.655 | 12.597 | 8.414 | 0.057 | 0.030 | 0.056 |
| AUC C-peptide 0-30/AUC glucose 0-30 | 186.671 | 202.387 | 206.219 | 14.797 | 4.211 | 2.180 | 0.021 | 0.015 | 0.142 |
| AUC C-peptide 0-120/AUC glucose 0-120 | 285.187 | 322.346 | 325.078 | 17.429 | 5.675 | 2.905 | 0.019 | 0.013 | 0.160 |
| Insulin 30 [pmol/] | 440.271 | 525.781 | 544.490 | 56.206 | 22.532 | 11.320 | 0.028 | 0.020 | 0.164 |
| Proinsulin 0 [pmol/l] | 5.118 | 6.250 | 5.578 | 0.745 | 0.394 | 0.164 | -0.040 | 0.044 | 0.359 |
| Proinsulin 0/Insulin 0 | 0.1140 | 0.1338 | 0.1110 | 0.0221 | 0.0131 | 0.0043 | -0.0265 | 0.0446 | 0.5525 |
| AUC proinsulin 0-120/AUC insulin 0-120 | 0.0555 | 0.0489 | 0.0472 | 0.0070 | 0.0023 | 0.0016 | -0.0052 | 0.0360 | 0.8855 |
| AUC proinsulin 60-120/AUC insulin 60-120 | 0.0730 | 0.0598 | 0.0591 | 0.0093 | 0.0028 | 0.0018 | 0.0058 | 0.0376 | 0.8770 |

|  |  |  |  |  |  |  |  |  |  |
| --- | --- | --- | --- | --- | --- | --- | --- | --- | --- |
|  | CC | CA | AA | CC | CA | AA |  |  |  |
| **GLIS3 rs7034200** | 464 | 885 | 432 |  |  |  |  |  |  |
| Glucose 0 [mmol/l] | 5.087 | 5.162 | 5.157 | 0.025 | 0.019 | 0.027 | 0.008 | 0.003 | 0.015 |
| Glucose 120 [mmol/l] | 6.344 | 6.399 | 6.349 | 0.075 | 0.054 | 0.082 | 0.001 | 0.008 | 0.907 |
| Insulin 0 [pmol/l] | 66.747 | 71.403 | 67.986 | 2.500 | 2.031 | 2.742 | 0.006 | 0.016 | 0.723 |
| ISI-Matsuda [U/l] | 15.418 | 15.112 | 15.493 | 0.464 | 0.361 | 0.513 | -0.013 | 0.017 | 0.446 |
| HOMA-IR [U] | 2.2377 | 2.4284 | 2.3122 | 0.0913 | 0.0737 | 0.1008 | 0.0133 | 0.0169 | 0.4315 |
| AUC insulin 0-30/AUC glucose 0-30 | 44.016 | 45.166 | 42.725 | 1.435 | 1.099 | 1.610 | -0.036 | 0.013 | 0.008 |
| Insulinogenic Index | 148.185 | 148.145 | 151.315 | 11.766 | 12.062 | 6.367 | -0.033 | 0.021 | 0.113 |
| AUC C-peptide 0-30/AUC glucose 0-30 | 205.644 | 207.522 | 199.055 | 3.675 | 2.767 | 3.892 | -0.022 | 0.010 | 0.028 |
| AUC C-peptide 0-120/AUC glucose 0-120 | 324.368 | 325.641 | 319.129 | 4.919 | 3.650 | 5.311 | -0.014 | 0.009 | 0.131 |
| Insulin 30 [pmol/] | 532.364 | 550.011 | 518.489 | 18.755 | 14.388 | 20.775 | -0.037 | 0.014 | 0.010 |
| Proinsulin 0 [pmol/l] | 5.746 | 5.745 | 5.653 | 0.332 | 0.215 | 0.297 | 0.032 | 0.031 | 0.294 |
| Proinsulin 0/Insulin 0 | 0.1254 | 0.1132 | 0.1134 | 0.0125 | 0.0050 | 0.0078 | 0.0364 | 0.0310 | 0.2404 |
| AUC proinsulin 0-120/AUC insulin 0-120 | 0.0457 | 0.0465 | 0.0527 | 0.0020 | 0.0018 | 0.0033 | 0.0616 | 0.0249 | 0.0136 |
| AUC proinsulin 60-120/AUC insulin 60-120 | 0.0575 | 0.0580 | 0.0651 | 0.0026 | 0.0021 | 0.0037 | 0.0602 | 0.0261 | 0.0212 |
|  |  |  |  |  |  |  |  |  |  |
|  | TT | TC | CC | TT | TC | CC |  |  |  |
| **PROX1 rs340874** | 400 | 844 | 530 |  |  |  |  |  |  |
| Glucose 0 [mmol/l] | 5.076 | 5.170 | 5.146 | 0.027 | 0.019 | 0.025 | 0.007 | 0.003 | 0.023 |
| Glucose 120 [mmol/l] | 6.297 | 6.375 | 6.426 | 0.081 | 0.056 | 0.072 | 0.013 | 0.008 | 0.086 |
| Insulin 0 [pmol/l] | 69.070 | 70.996 | 66.854 | 2.863 | 2.118 | 2.277 | 0.020 | 0.016 | 0.191 |
| ISI-Matsuda [U/l] | 15.807 | 15.101 | 15.167 | 0.532 | 0.362 | 0.448 | -0.036 | 0.017 | 0.035 |
| HOMA-IR [U] | 2.3156 | 2.4230 | 2.2574 | 0.1026 | 0.0779 | 0.0828 | 0.0275 | 0.0167 | 0.0991 |
| AUC insulin 0-30/AUC glucose 0-30 | 45.592 | 44.157 | 43.379 | 1.670 | 1.140 | 1.333 | -0.026 | 0.013 | 0.050 |
| Insulinogenic Index | 155.845 | 147.260 | 146.259 | 12.997 | 12.615 | 6.228 | -0.038 | 0.021 | 0.065 |
| AUC C-peptide 0-30/AUC glucose 0-30 | 211.421 | 204.502 | 200.078 | 4.201 | 2.829 | 3.320 | -0.027 | 0.010 | 0.006 |
| AUC C-peptide 0-120/AUC glucose 0-120 | 329.055 | 322.990 | 319.825 | 5.619 | 3.753 | 4.476 | -0.016 | 0.009 | 0.085 |
| Insulin 30 [pmol/] | 546.965 | 538.329 | 529.115 | 21.766 | 14.862 | 17.407 | -0.020 | 0.014 | 0.149 |
| Proinsulin 0 [pmol/l] | 5.883 | 5.778 | 5.522 | 0.356 | 0.223 | 0.270 | 0.002 | 0.030 | 0.952 |
| Proinsulin 0/Insulin 0 | 0.1207 | 0.1179 | 0.1115 | 0.0122 | 0.0060 | 0.0074 | 0.0126 | 0.0308 | 0.6829 |
| AUC proinsulin 0-120/AUC insulin 0-120 | 0.0443 | 0.0500 | 0.0472 | 0.0018 | 0.0023 | 0.0018 | 0.0347 | 0.0247 | 0.1602 |
| AUC proinsulin 60-120/AUC insulin 60-120 | 0.0565 | 0.0615 | 0.0590 | 0.0025 | 0.0026 | 0.0024 | 0.0340 | 0.0258 | 0.1867 |
|  |  |  |  |  |  |  |  |  |  |
|  | GG | GA | AA | GG | GA | AA |  |  |  |
| **C2CD4B rs11071657** | 222 | 826 | 730 |  |  |  |  |  |  |
| Glucose 0 [mmol/l] | 5.085 | 5.127 | 5.172 | 0.034 | 0.019 | 0.021 | 0.003 | 0.003 | 0.423 |
| Glucose 120 [mmol/l] | 6.327 | 6.373 | 6.380 | 0.113 | 0.056 | 0.061 | -0.005 | 0.008 | 0.567 |
| Insulin 0 [pmol/l] | 67.910 | 67.360 | 71.630 | 3.613 | 2.029 | 2.135 | 0.012 | 0.017 | 0.456 |
| ISI-Matsuda [U/l] | 15.997 | 15.556 | 14.762 | 0.705 | 0.370 | 0.383 | -0.021 | 0.018 | 0.239 |
| HOMA-IR [U] | 2.2761 | 2.2700 | 2.4456 | 0.1285 | 0.0726 | 0.0795 | 0.0151 | 0.0178 | 0.3963 |
| AUC insulin 0-30/AUC glucose 0-30 | 44.850 | 43.694 | 44.685 | 2.138 | 1.138 | 1.192 | -0.016 | 0.014 | 0.259 |
| Insulinogenic Index | 179.303 | 137.655 | 152.403 | 13.546 | 12.508 | 8.124 | -0.028 | 0.022 | 0.205 |
| AUC C-peptide 0-30/AUC glucose 0-30 | 206.407 | 204.017 | 205.656 | 5.277 | 2.822 | 3.041 | -0.012 | 0.011 | 0.279 |
| AUC C-peptide 0-120/AUC glucose 0-120 | 324.481 | 321.234 | 326.407 | 7.391 | 3.687 | 4.089 | -0.002 | 0.010 | 0.863 |
| Insulin 30 [pmol/] | 528.304 | 532.305 | 545.602 | 25.806 | 14.906 | 15.757 | -0.012 | 0.015 | 0.438 |
| Proinsulin 0 [pmol/l] | 5.756 | 5.664 | 5.770 | 0.499 | 0.217 | 0.244 | -0.009 | 0.032 | 0.787 |
| Proinsulin 0/Insulin 0 | 0.1167 | 0.1152 | 0.1180 | 0.0099 | 0.0055 | 0.0087 | -0.0059 | 0.0328 | 0.8577 |
| AUC proinsulin 0-120/AUC insulin 0-120 | 0.0464 | 0.0469 | 0.0494 | 0.0030 | 0.0014 | 0.0026 | 0.0231 | 0.0264 | 0.3812 |
| AUC proinsulin 60-120/AUC insulin 60-120 | 0.0586 | 0.0578 | 0.0620 | 0.0040 | 0.0017 | 0.0029 | 0.0229 | 0.0275 | 0.4048 |
